# Alpha-Synuclein Amyloid Aggregation Is Inhibited by Sulfated Aromatic Polymers and Pyridinium Polycation

**DOI:** 10.3390/polym12030517

**Published:** 2020-02-28

**Authors:** Pavel Semenyuk, Lidia Kurochkina, Kseniya Barinova, Vladimir Muronetz

**Affiliations:** 1 Belozersky Institute of Physico-Chemical Biology, Lomonosov Moscow State University, 119234 Moscow, Russia; lpk56@mail.ru (L.K.); kmerkushina@gmail.com (K.B.); vimuronets@belozersky.msu.ru (V.M.); 2 Faculty of Bioengineering and Bioinformatics, Lomonosov Moscow State University, 119234 Moscow, Russia

**Keywords:** polyelectrolyte, sulfated polymers, alpha-synuclein, amyloid aggregation, protein aggregation, artificial chaperone

## Abstract

The effect of a range of synthetic charged polymers on alpha-synuclein aggregation and amyloid formation was tested. Sulfated aromatic polymers, poly(styrene sulfonate) and poly(anethole sulfonate), have been found to suppress the fibril formation. In this case, small soluble complexes, which do not bind with thioflavin T, have been formed in contrast to the large stick-type fibrils of free alpha-synuclein. Sulfated polysaccharide (dextran sulfate), as well as sulfated vinylic polymer (poly(vinyl sulfate)) and polycarboxylate (poly(methacrylic acid)), enhanced amyloid aggregation. Conversely, pyridinium polycation, poly(N-ethylvinylpyridinium), switched the mechanism of alpha-synuclein aggregation from amyloidogenic to amorphous, which resulted in the formation of large amorphous aggregates that do not bind with thioflavin T. The obtained results are relevant as a model of charged macromolecules influence on amyloidosis development in humans. In addition, these results may be helpful in searching for new approaches for synucleinopathies treatment with the use of natural polymers.

## 1. Introduction

Alpha-synuclein is the main protein involved in the development and progression of various synucleinopathies [1,2,3], especially Parkinson’s disease [4,5,6,7]. It can form two different types of amyloid structures: toxic oligomers and amyloid fibrils [8,9]. The pathological role of the latter seems to be disputable since the deposition of the amyloid fibrils and the formation of so-called Lewy bodies can serve as a protective mechanism against the toxic effect of alpha-synuclein oligomers toward nerve cells [10,11]. Despite numerous studies on the amyloid transformation of alpha-synuclein, the mechanism underlying this process and approaches for prevention synucleinopathies remain unclear.

Several approaches for the prevention and treatment of amyloidosis, including synucleinopathies, were proposed. For example, the use of molecular chaperones is a promising approach to treat amyloidosis [12,13]. Thus, overproduction of Hsp70 and Hsp104 inhibits the propagation of yeast prion and huntingtin [14,15,16]. The therapeutic effect of Hsp70 has also been shown for Alzheimer’s disease [17]. Despite a high complexity of the chaperone system and sometimes opposite effects of different chaperones on amyloid aggregation [16,18], few chaperone-based treatments were suggested as a promising platform for the treatment of diseases associated with proteinopathies [19].

Synthetic charged polymers can be considered as artificial chaperones, which can protect enzymes against amorphous aggregation and inactivation [20,21,22,23]. The efficiency of sulfated polymers can be even higher than the activity of natural chaperones [24]. Furthermore, it was shown that polymers are able to extract the enzyme from pre-formed aggregates with its subsequent partial reactivation in solution [25], as well as in a microgel form with subsequent reactivation using cyclodextrin [26,27]. The action and efficiency depend on many factors such as polyelectrolyte charge [28], the nature of its charged groups [29], the hydrophobicity of the polymer [30], degree of polymerization [21] and other factors [31,32].

Interaction of some polyanions, sulfated glycosaminoglycans, with amyloidogenic proteins was studied previously [33]. Thus, heparin and heparan sulfate were shown to induce amyloid aggregation of apolipoprotein A-I [34,35] and serum amyloid A [36]. Noteworthy, electrostatic interactions are of special importance for amyloid aggregation [37]. The results of the inhibition of amyloid aggregation by synthetic polymers have also been described. For example, cationic pyridylphenylene [38,39] and polyamidoamine [40] dendrimers exhibited anti-amyloid activity. Heparin was shown to modulate the aggregation of amyloid-beta peptide [41]. In addition, we have recently found that the treatment of amyloid fibrils of ovine prion protein with poly(styrene sulfonate) enhances its proteolytic degradation [42]. As for alpha-synuclein, some sulfated polysaccharides stimulated its fibrillation (heparin, heparin sulfate, etc.), but some of them did not (for example, keratan sulfate) [43,44]. Thus, there is extensive, but controversial and unsystematic information about the influence of biopolymers on the amyloid transformation of amyloidogenic proteins and, especially, little information about alpha-synuclein transformation.

In the present work, we examined the influence of a set of synthetic polyanions and polycations on the amyloid aggregation of human alpha-synuclein, to reveal the effect of different biomolecules on its amyloid conversion. The understanding of this effect is required to select biopolymers that can be used as anti-aggregation agents for the prevention and treatment of synucleinopathies. The polymers differed in the nature of the charged groups, in their structure and degree of polymerization. We measured the kinetics of amyloid aggregation, and also determined the size and structure of the particles formed. Sulfated aromatic polyanions, poly(styrene sulfonate) and poly(anethole sulfonate), have been found to inhibit fibril formation unlike other polyanions. Pyridinium polycation, poly(N-ethylvinylpyridinium), switched the mechanism of alpha-synuclein aggregation from amyloidogenic to amorphous.

## 2. Materials and Methods

### 2.1. Purification of Human Recombinant Alpha-Synuclein

Human recombinant alpha-synuclein was isolated from the *E. coli* producer strain using acid precipitation of contaminating proteins with subsequent sulfate ammonium precipitation, and the final purification on a thiol-Sepharose column [45]. Briefly, the pH value of the original cell extract was adjusted to 2.8 by adding 9% HCl, and the aggregated proteins were removed by centrifugation (15000× *g*, 5 min, 4 °C). The pH value of the supernatant was adjusted to 7.5 with 1 M potassium phosphate solution, pH 11. Then, sulfate ammonium was added to the supernatant to reach 40% saturation, and the suspension was left overnight at 4 °C. The resulting preparation of human recombinant alpha-synuclein contained an admixture of Tyr136Cys substituted alpha-synuclein that was produced due to the translational error. Tyr136Cys substituted alpha-synuclein was removed on a thiol-Sepharose column.

### 2.2. Polymers

**Scheme 1:**
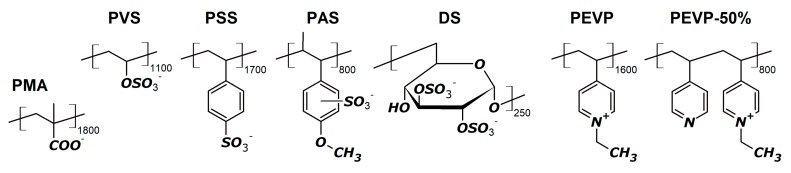
Structures of the tested polyanions and polycations.

Structures of the used polymers are shown in Scheme 1. Samples of sodium poly(styrene sulfonate) (PSS) with a polymerization degree of 1700 (350 kDa), sodium poly(anethole sulfate) (PAS) with an approximate polymerization degree of 800 (200 kDa), sodium dextran sulfate (DS) with a molecular mass of 100 kDa (approximately 250 repeated units and 600 charged groups), potassium poly(vinyl sulfate) (PVS) with an approximate polymerization degree of 1100 (180 kDa) and polymethacrylic (PMA) acid with an approximate polymerization degree of 1800 (150 kDa) were purchased from Sigma-Aldrich (St. Louis, MO, USA). Poly(N-ethyl-4-vinylpyridinium) bromide (PEVP) with a polymerization degree of 1600 (340 kDa), as well as a statistical copolymer of N-ethyl-4-vinylpyridinium bromide and 4-vinylpyridine (PEVP-50%) were synthesized by alkylation of the poly(4-vinylpyridinium) sample with ethyl bromide as described in [46] and kindly provided by Prof. Vladimir Izumrudov from Lomonosov Moscow State University (Moscow, Russia). Polyelectrolyte concentrations were expressed in the terms of the molar concentration of charged groups excepting PEVP-50%, in which the molar concentration of chains was the same as PEVP.

### 2.3. Aggregation Assay

Amyloid aggregation of alpha-synuclein was achieved by intense shaking of its solution in 10 mM potassium phosphate buffer, pH 4.0, containing 137 mM sodium chloride and 2.7 mM potassium chloride at 37 °C. Two series of experiments with no significant difference in aggregation levels were performed: in 96-well FLUOTRAC 200 black immunology plates (100 µL) and in glass vials (300 µL) as described in [47]. The size of the formed particles and scattering intensity were estimated by dynamic light scattering measurement on a Zetasizer NanoZS (Malvern Instruments, Worcestershire, UK) instrument, using a scattering angle of 173° and wavelength of 633 nm, without centrifuging the samples. Amount of amyloid structures was determined from the increase of thioflavin T fluorescence at 480 nm after excitation at 435 nm. Measurements were performed in 96-well FLUOTRAC 200 black immunology plates (Greiner, Kremsmünster, Austria) using a VICTOR X5 Light Plate Reader (PerkinElmer, Waltham, MA, USA). Protein concentration was 0.4 mg/mL (29 µM), concentration of polymers’ charged groups was 5 mM (that is approximately 3 µM, 4.5 µM, 3 µM, 6 µM, 9 µM, 3 µM and 3 µM for PMA, PVS, PSS, PAS, DS, PEVP and PEVP-50%, respectively), thioflavin T (ThT) was added 15 min before the first measurement in 10-fold molar excess. The values of ThT fluorescence in the absence of protein (i.e., controls for free polymers) were subtracted from the values obtained for the main mixtures of alpha-synuclein and corresponding polymers. The final curves are the average of three independent experiments.

### 2.4. Circular Dichroism Spectroscopy

Circular dichroism measurements were performed using a Chiroscan CD spectrometer (Applied Photophysics, Leatherhead, Surrey, UK) in a 0.1-mm-pathlength cell. Spectra were recorded in the range of 195–260 nm and were baseline corrected by subtracting the buffer spectrum. Each point was measured for 1 s. For each system, 3–6 measurements were performed and averaged. The observed value was converted into mean residue molar ellipticity.

### 2.5. Transmission Electron Microscopy

The samples of free alpha-synuclein without additions, as well as its mixtures with polymers, were incubated in 250-µL glass vials with intense shaking at 37 °C for 36 h. Protein and polymer concentrations were 0.4 mg/mL (29 µM of alpha-synuclein monomers) and 5 mM (in terms of charged groups), respectively. The same buffer system as in other experiments was used. Immediately before application, the samples were diluted with pure water in the ratio of 1:1. The samples were adsorbed onto a Formvar film attached to 300-mesh copper grids and contrasted with 1% uranyl acetate aqueous solution. The specimens were observed in a JEOL JEM-1400 electron microscope (JEOL, Akishima, Japan) at 100 kV × 40,000 magnification. The length and diameter of fibrils were determined from the images using ImageJ (https://imagej.nih.gov/ij/) software.

## 3. Results and Discussion

**Figure 1:**
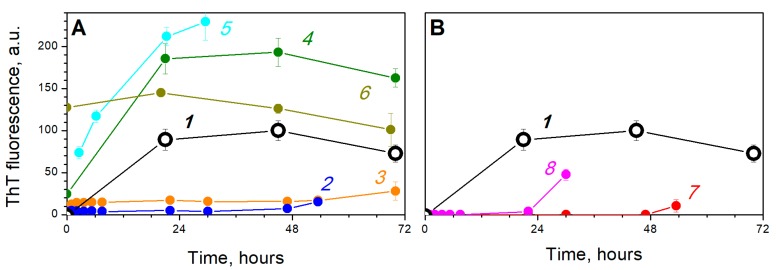
Kinetics of fibril formation detected by thioflavin T (ThT) fluorescence assay. (**A**) Free alpha-synuclein (1), alpha-synuclein in the presence of 5 mM poly(styrene sulfonate) (PSS) (2), poly(anethole sulfate) (PAS) (3), dextran sulfate (DS) (4), poly(vinyl sulfate) (PVS) (5) and polymethacrylic acid (PMA) (6). (**B**) Free alpha-synuclein (1), alpha-synuclein in the presence of 5 mM poly(N-ethyl-4-vinylpyridinium) bromide (PEVP) (7) and PEVP-50% (8). Fluorescence intensity is presented as a percentage of the maximum for free alpha-synuclein. The values of ThT fluorescence in the presence of polymers without protein are subtracted.

**Figure 2:**
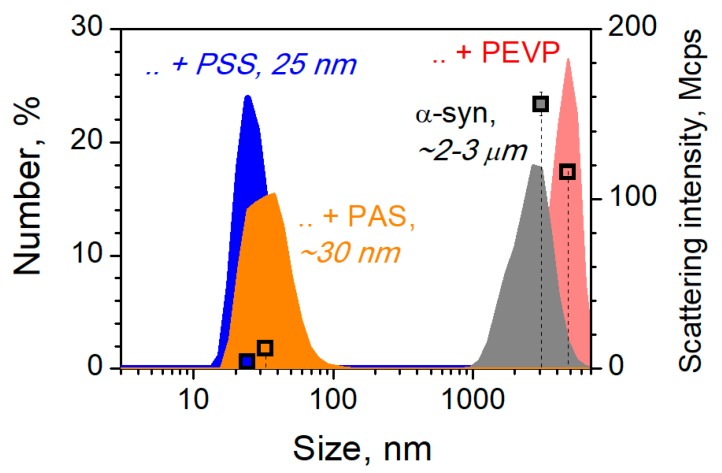
Hydrodynamic diameter of particles formed after 2-day incubation of alpha-synuclein in the absence (gray) and in the presence of 5 mM PSS (blue), PAS (orange) or PEVP (pink). Squares represent scattering intensity, the color and position of each square coincide to those of the corresponding hydrodynamic diameter distribution peak.

First, we studied the kinetics of amyloid aggregation of alpha-synuclein without additions and in the presence of polymers monitoring the binding with thioflavin T (ThT). Structures of the used polymers are shown in Scheme 1: polyanions sodium poly(styrene sulfonate) (PSS) with a polymerization degree of 1700 (350 kDa), sodium poly(anethole sulfate) (PAS) with an approximate polymerization degree of 800 (200 kDa), sodium dextran sulfate (DS) with a molecular mass of 100 kDa (approximately 250 repeated units and 600 charged groups), potassium poly(vinyl sulfate) (PVS) with an approximate polymerization degree of 1100 (180 kDa) and polymethacrylic acid (PMA) with an approximate polymerization degree of 1800 (150 kDa); polycations poly(N-ethyl-4-vinylpyridinium) bromide (PEVP) with a polymerization degree of 1600 (340 kDa) and a statistical copolymer of N-ethyl-4-vinylpyridinium bromide and 4-vinylpyridine (PEVP-50%). All sulfated polymers bear a strong negative charge under the selected conditions; PMA is partially negatively charged; both polycations are strongly positively charged; alpha-synuclein is slightly positively charged since its pI is 4.7. Free alpha-synuclein formed amyloid structures and reached a plateau in less than one day (Figure 1A, line 1). In the presence of both sulfated aromatic polymers, poly(styrene sulfonate) and poly(anethole sulfonate), ThT fluorescence did not increase from the initial level for 2–3 days (Figure 1A, lines 2–3). According to visual observations, the mixtures of alpha-synuclein with PSS and PAS remained clear solutions, unlike the sample of free alpha-synuclein, which became relatively turbid after 15–20 h because of fibril formation. Thus, scattering intensities at 173° were 4 ± 0.2, 12 ± 3 and 156 ± 7 Mcps for alpha-synuclein incubated in the presence of PSS, PAS and in free form, respectively (Figure 2). On the contrary, dextran sulfate significantly enhanced the level of amyloid aggregation of alpha-synuclein (Figure 1A, line 4). It is consistent with the data of other research groups about the influence of sulfated polysaccharides, glycosaminoglycans, such as heparin, heparan sulfate, etc., on the amyloid transformation of alpha-synuclein and other amyloidogenic proteins [34,35,36,43]. Poly(vinyl sulfate) had a similar but even more pronounced effect (Figure 1A, line 5). A similar picture was observed in the case of the carboxylic polymer, poly(methacrylate). The mixture of alpha-synuclein and PMA became turbid rapidly, then large flakes were formed, and ThT fluorescence increased significantly (Figure 1A, line 6). In other words, PMA did not inhibit alpha-synuclein aggregation.

Pyridinium polycation, poly(N-ethyl-4-vinylpyridinium) (PEVP), was found to inhibit ThT fluorescence growth (Figure 1B, line 7). However, the solution became turbid almost immediately after mixing indicating the formation of large aggregates. We also tested a partially alkylated poly(4-vinylpyridine) sample, PEVP-50%, in which only 50% of repeated units were charged. Its inhibiting efficiency toward amyloid fibrils formation was lower compared with PEVP (Figure 1B, line 8).

Thus, two sulfonated polymers, PAS and PSS, as well as polycations, PEVP and PEVP-50%, seem to inhibit amyloid fibrils formation, and therefore these systems were studied in details. The size of the particles formed over three days, which did not bind with ThT, was measured using dynamic light scattering. Small complexes with a hydrodynamic diameter of 25–30 nm were formed in the presence of both polyanions, PSS and PAS, (Figure 2). Of course, these values most likely do not reflect the actual particle size because of the non-spherical shape of the particles. However, comparing them with the values for fibrils formed by free alpha-synuclein (2–3 µm, see Figure 2, gray) makes the difference clear. A completely different picture was observed for the mixture of alpha-synuclein and polycation, PEVP. The extremely large particles were formed (Figure 2, pink). In other words, PEVP did not inhibit aggregation, but, on the contrary, increased the particle size. However, these large particles did not bind with ThT (Figure 1, line 7) suggesting their non-amyloid nature.

**Figure 3:**
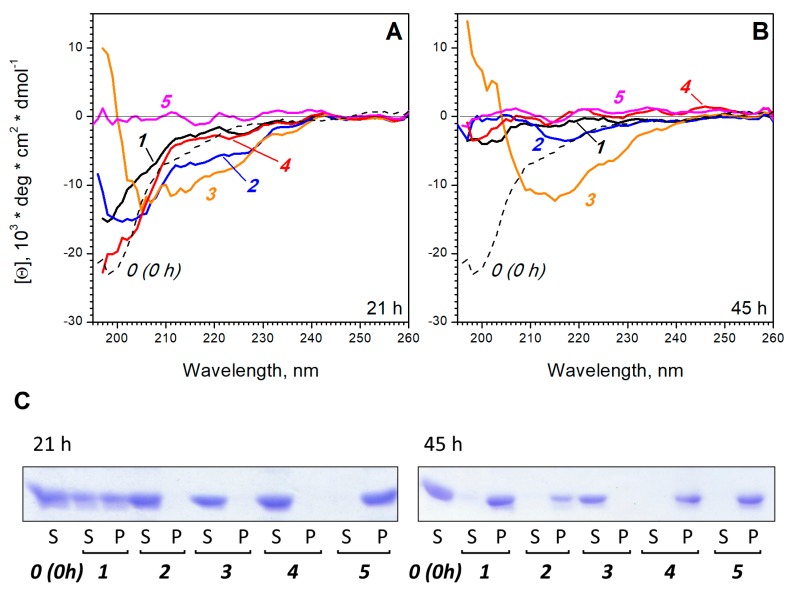
Circular dichroism spectra of intact alpha-synuclein monomers before incubation under fibrillation conditions (0) and after the incubation for 21 h (**A**) and 45 h (**B**) in free form (1) and in the presence of 5 mM PSS (2), PAS (3), PEVP (4) and PEVP-50% (5). (**C**) SDS-PAGE of supernatant (S) and pellet (P) fractions of the same samples 0–5 centrifuged for 10 min at 12,000× *g*.

For additional characterization of the influence of polymers on alpha-synuclein aggregation, circular dichroism measurements were performed (Figure 3). After one day of the incubation, the ellipticity in the spectrum region corresponding to random coil decreased, while the ellipticity in the region corresponding to β-structures increased (Figure 3A, lines 0 and 1), indicating an increase of the β-structure content [48] because of amyloid-like conversion. A more pronounced increase of the β-structure content was observed in the presence of PSS and, especially, PAS (Figure 3A, lines 2–3). After one more day of incubation, no ellipticity peaks were observed in the spectrum of free alpha-synuclein (Figure 3B, line 1) because of intensive aggregation and the formation of large particles that hinders measurement. SDS-PAGE of the supernatant and pellet fractions from the samples centrifuged for 10 min at 12,000× *g* also indicates aggregation of fibrils (Figure 3C). A similar effect was observed for the mixture of alpha-synuclein and both polycations (PEVP and PEVP-50%) (Figure 3B, lines 4–5, Figure 3C). On the contrary, β-structures were observed in the presence of PAS, suggesting that PAS induced amyloid conversion of alpha-synuclein, but inhibited aggregation (Figure 3B,C). This suggestion agrees well with the data of ThT fluorescence measurements: the initial level of ThT binding by particles formed in the presence of PAS was higher than that in the case of free alpha-synuclein, but it did not change for 2–3 days unlike free alpha-synuclein (Figure 1). PSS demonstrated similar behavior, but the content of β-structures (Figure 3B, line 2), as well as the initial ThT binding level (Figure 1), were much lower compared to PAS.

**Figure 4:**
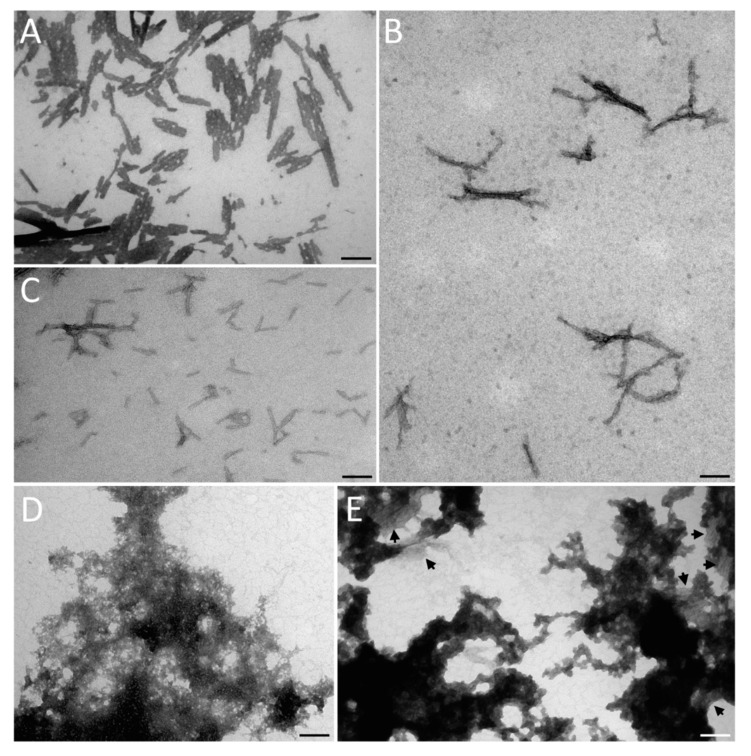
Electron micrographs of amyloid fibrils of free alpha-synuclein (**A**) and particles formed after 1.5-days of incubation of alpha-synuclein with 5 mM PAS (**B**), PSS (**C**), PEVP (**D**) and PEVP-50% (**E**). Black arrows in (E) show amyloid fibrils. Scale bar is 100 nm in all panels.

Electron microscopy data corroborated our suggestions. Thus, free alpha-synuclein efficiently formed rod-like tightly packed fibrils, sticking to each other by lateral sides (Figure 4A). The length and diameter of individual fibrils were estimated to be 88 ± 23 nm and 10.8 ± 1.0 nm, respectively. Such fibrils were described in [49] as one of two types of alpha-synuclein fibrils, short rod-like and long flexible fibrils. A completely different picture was observed for the mixture of alpha-synuclein and sulfated aromatic polymers, PSS and PAS (Figure 4B,C). In the presence of PAS, the particles similar to fibrils were almost single and did not stick to each other (Figure 4B). The length of individual fibrils increased up to 155 ± 50 nm, although the diameter did not change (10.4 ± 1.9 nm). Noteworthy, the particle size in the presence of PAS was lower than in the absence of polymer, since the sticking seems to be blocked, suggesting a partial suppression of amyloid aggregation. In the presence of PSS, much smaller particles were formed (Figure 4C). They looked like a kind of short fibrils that did not interact with each other. The average length of such particles was 60 ± 14 nm, which was lower compared to the control fibrils, though the diameter did not change (10.6 ± 0.7 nm). Summarizing the results mentioned above, we can conclude that both sulfated aromatic polymers pronouncedly influence on alpha-synuclein aggregation, and PSS significantly suppresses the formation of amyloid fibrils.

As for the polycation, the complexes formed in the presence of PEVP were large and did not bind with ThT demonstrating an amorphous structure (Figure 4D). In other words, addition of PEVP switched the mechanism of alpha-synuclein aggregation from amyloidogenic to amorphous: the turbid system contained large complexes, which did not bind ThT. Noteworthy, the anti-aggregation activity of partially alkylated polymer, PEVP-50%, was less efficient. Although the large aggregates formed in the presence of PEVP-50% looked roughly similar to the aggregates formed in the presence of PEVP, they were denser and contained a significant amount of tightly packed amyloid fibrils (Figure 4E, black arrows). This finding agrees well with the ThT fluorescence data: despite PEVP-50% retarding the amyloid fibrils formation, after 36 h incubation, the amount of amyloid structures became pronounced (Figure 1B, line 8).

**Scheme 2:**
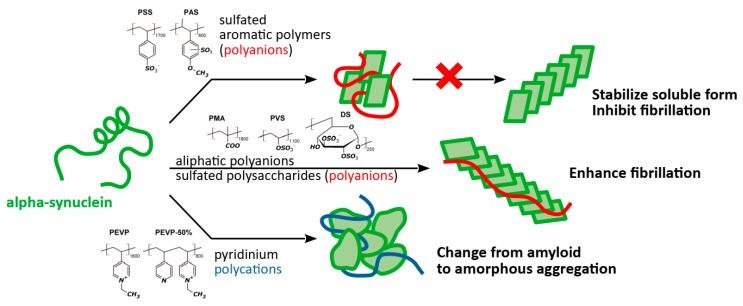
Schematic summary of influence of the tested polymers on amyloid aggregation of alpha-synuclein.

Summarizing the results (Scheme 2), we conclude that sulfated aromatic polymers, especially poly(styrene sulfonate), significantly suppress alpha-synuclein fibrillation. Since the initial ThT fluorescence in the mixture of alpha-synuclein and PAS was slightly higher than that of free alpha-synuclein, binding to the polyanion probably altered the protein conformation and thus induced amyloid conversion of the initial monomeric forms. This hypothesis is also corroborated with CD data, which indicated a high content of β-structures in alpha-synuclein samples incubated with PAS. However, sulfated polymers, which may be considered as artificial chaperones [24], appear to retard protein–protein interactions, and therefore the fibril formation is inhibited. This statement was confirmed by transmission electron microscopy data: in the presence of both sulfated polymers, PSS and PAS, we observed no sticking of fibril-like structures to each other in contrast to free alpha-synuclein fibrils, which efficiently stuck to each other, forming large aggregates (Figure 4). Other polyanions, including polycarboxylate, did not protect alpha-synuclein against amyloid aggregation. This finding agrees well with the data that chaperone-like activity of phosphate- or carboxylate-based polyanions is lower than that of sulfated and sulfonated polymers [29]. At the same time, the polycation, PEVP, was found to switch the mechanism of aggregation from amyloidogenic to amorphous. Thus, we conclude that charged polymers, especially sulfated polymers, may be considered as a new class of prospective anti-amyloid agents. Moreover, they can be created on the basis of natural polymers, which will facilitate their therapeutic usage.

The obtained results are of special interest as a model of interactions that occur in humans during the development and propagation of amyloidosis associated with alpha-synuclein. Human cells contain many charged polymers, including heparan sulfate and other sulfated glycosaminoglycans [37]. Their influence on amyloidogenic proteins, including alpha-synuclein, was studied in a few works [34,35,36,43]. According to published results and our data on a similar polymer, dextran sulfate, sulfated polysaccharides can enhance or even induce amyloid transformation and aggregation of amyloidogenic proteins. However, our results show that this effect is not the same for all polyanions. Anyway, interaction with polyanions and polycations seems to be an important factor (and, maybe, a key factor), influencing the amyloidosis development, and it should be taken into account in studies. Furthermore, synthetic polymers can be considered as a simplified model of proteins since many proteins have charged regions on their surfaces. Non-specific interactions with these proteins may affect alpha-synuclein aggregation, as it was, for example, in the case of the glycolytic enzyme, glyceraldehyde-3-phosphate dehydrogenase: binding with the anion-binding groove of the enzyme inhibited the amyloid aggregation of alpha-synuclein [47].

Thus, on the basis of the obtained results, it is possible to predict the effect of the natural polymers present in the cell on the amyloid transformation of alpha-synuclein and, therefore, on the development of synucleinopathies. The possibility to predict the effects of various posttranslational modifications (glycation, oxidation, phosphorylation, sulfation, etc.) of both alpha-synuclein and its partner proteins on the amyloidosis genesis of this type is of special significance [50]. In addition, a comparison of the effects of polymers of different nature on amyloidogenic transformation provides a fundamental basis for the rational selection of potential anti-amyloid agents, both among synthetic and natural compounds.

## References

[1] Sanford A.M. (2018). Lewy Body Dementia. Clin. Geriatr. Med..

[2] Giasson B.I., Duda J.E., Murray I.V., Chen Q., Souza J.M., Hurtig H.I., Ischiropoulos H., Trojanowski J.Q., Lee V.M. (2000). Oxidative damage linked to neurodegeneration by selective alpha-synuclein nitration in synucleinopathy lesions. Science.

[3] Barker R.A., Williams-Gray C.H. (2016). Review: The spectrum of clinical features seen with alpha synuclein pathology. Neuropathol. Appl. Neurobiol..

[4] Uversky V.N. (2007). Neuropathology, biochemistry, and biophysics of α-synuclein aggregation. J. Neurochem..

[5] Spillantini M.G., Schmidt M.L., Lee V.M., Trojanowski J.Q., Jakes R., Goedert M. (1997). Alpha-synuclein in Lewy bodies. Nature.

[6] Perez R.G., Hastings T.G. (2004). Could a loss of alpha-synuclein function put dopaminergic neurons at risk?. J. Neurochem..

[7] O’Leary E.I., Lee J.C. (2018). Interplay between α-synuclein amyloid formation and membrane structure. Biochim. Biophys. Acta Proteins Proteom..

[8] Iljina M., Hong L., Horrocks M.H., Ludtmann M.H., Choi M.L., Hughes C.D., Ruggeri F.S., Guilliams T., Buell A.K., Lee J.-E. (2017). Nanobodies raised against monomeric ɑ-synuclein inhibit fibril formation and destabilize toxic oligomeric species. BMC Biol..

[9] Chen S.W., Drakulic S., Deas E., Ouberai M., Aprile F.A., Arranz R., Ness S., Roodveldt C., Guilliams T., De-Genst E.J. (2015). Structural characterization of toxic oligomers that are kinetically trapped during α-synuclein fibril formation. Proc. Natl. Acad. Sci. USA.

[10] Soto C., Estrada L.D. (2008). Protein misfolding and neurodegeneration. Arch. Neurol..

[11] Bengoa-Vergniory N., Roberts R.F., Wade-Martins R., Alegre-Abarrategui J. (2017). Alpha-synuclein oligomers: A new hope. Acta Neuropathol..

[12] Paul S., Mahanta S. (2014). Association of heat-shock proteins in various neurodegenerative disorders: Is it a master key to open the therapeutic door?. Mol. Cell. Biochem..

[13] Ben-Zvi A.P., Goloubinoff P. (2001). Review: Mechanisms of Disaggregation and Refolding of Stable Protein Aggregates by Molecular Chaperones. J. Struct. Biol..

[14] Chernoff Y.O., Lindquist S.L., Ono B., Inge-Vechtomov S.G., Liebman S.W. (1995). Role of the chaperone protein Hsp104 in propagation of the yeast prion-like factor [psi+]. Science.

[15] Krobitsch S., Lindquist S. (2000). Aggregation of huntingtin in yeast varies with the length of the polyglutamine expansion and the expression of chaperone proteins. Proc. Natl. Acad. Sci. USA.

[16] Newnam G.P., Wegrzyn R.D., Lindquist S.L., Chernoff Y.O. (1999). Antagonistic Interactions between Yeast Chaperones Hsp104 and Hsp70 in Prion Curing. Mol. Cell. Biol..

[17] Bobkova N.V., Garbuz D.G., Nesterova I., Medvinskaya N., Samokhin A., Alexandrova I., Yashin V., Karpov V., Kukharsky M.S., Ninkina N.N. (2014). Therapeutic Effect of Exogenous Hsp70 in Mouse Models of Alzheimer’s Disease. J. Alzheimer’s Dis..

[18] Kiselev G.G., Naletova I.N., Sheval E.V., Stroylova Y.Y., Schmalhausen E.V., Haertlé T., Muronetz V.I. (2011). Chaperonins induce an amyloid-like transformation of ovine prion protein: The fundamental difference in action between eukaryotic TRiC and bacterial GroEL. Biochim. Biophys. Acta.

[19] Lazarev V.F., Mikhaylova E.R., Guzhova I.V., Margulis B.A. (2017). Possible Function of Molecular Chaperones in Diseases Caused by Propagating Amyloid Aggregates. Front. Neurosci..

[20] Martin N., Ruchmann J., Tribet C. (2015). Prevention of Aggregation and Renaturation of Carbonic Anhydrase via Weak Association with Octadecyl- or Azobenzene-Modified Poly(acrylate) Derivatives. Langmuir.

[21] Shalova I.N., Asryants R.A., Sholukh M.V., Saso L., Kurganov B.I., Muronetz V.I., Izumrudov V.A. (2005). Interaction of polyanions with basic proteins, 2(a): Influence of complexing polyanions on the thermo-aggregation of oligomeric enzymes. Macromolar Bioscience.

[22] Shalova I.N., Naletova I.N., Saso L., Muronetz V.I., Izumrudov V.A. (2007). Interaction of polyelectrolytes with proteins, 3. Influence of complexing polycations on the thermoaggregation of oligomeric enzymes. Macromol. Biosci..

[23] Semenyuk P., Tiainen T., Hietala S., Tenhu H., Aseyev V., Muronetz V. (2019). Artificial chaperones based on thermoresponsive polymers recognize the unfolded state of the protein. Int. J. Biol. Macromol..

[24] Semenyuk P.I., Kurochkina L.P., Gusev N.B., Izumrudov V.A., Muronetz V.I. (2017). Chaperone-like activity of synthetic polyanions can be higher than the activity of natural chaperones at elevated temperature. Biochem. Biophys. Res. Commun..

[25] Semenyuk P.I., Moiseeva E.V., Stroylova Y.Y., Lotti M., Izumrudov V.A., Muronetz V.I. (2015). Sulfated and sulfonated polymers are able to solubilize efficiently the protein aggregates of different nature. Arch. Biochem. Biophys..

[26] Nomura Y., Sasaki Y., Takagi M., Narita T., Aoyama Y., Akiyoshi K. (2005). Thermoresponsive Controlled Association of Protein with a Dynamic Nanogel of Hydrophobized Polysaccharide and Cyclodextrin:  Heat Shock Protein-Like Activity of Artificial Molecular Chaperone. Biomacromolecules.

[27] Takahashi H., Sawada S., Akiyoshi K. (2011). Amphiphilic Polysaccharide Nanoballs: A New Building Block for Nanogel Biomedical Engineering and Artificial Chaperones. ACS Nano.

[28] Sofronova A.A., Izumrudov V.A., Muronetz V.I., Semenyuk P.I. (2017). Similarly charged polyelectrolyte can be the most efficient suppressor of the protein aggregation. Polymer.

[29] Semenyuk P.I., Muronetz V.I., Haertlé T., Izumrudov V.A. (2013). Effect of poly (phosphate) anions on glyceraldehyde-3-phosphate dehydrogenase structure and thermal aggregation: Comparison with influence of poly(sulfoanions). Biochim. Biophys. Acta.

[30] Martin N., Ma D., Herbet A., Boquet D., Winnik F.M., Tribet C. (2014). Prevention of Thermally Induced Aggregation of IgG Antibodies by Noncovalent Interaction with Poly(acrylate) Derivatives. Biomacromolecules.

[31] Kayitmazer A.B., Seyrek E., Dubin P.L., Staggemeier B.A. (2003). Influence of Chain Stiffness on the Interaction of Polyelectrolytes with Oppositely Charged Micelles and Proteins. J. Phys. Chem. B.

[32] Seyrek E., Dubin P.L., Tribet C., Gamble E.A. (2003). Ionic Strength Dependence of Protein-Polyelectrolyte Interactions. Biomacromolecules.

[33] Ghosh D., Mehra S., Sahay S., Singh P.K., Maji S.K. (2017). α-synuclein aggregation and its modulation. Int. J. Biol. Macromol..

[34] Rosú S.A., Toledo L., Urbano B.F., Sanchez S.A., Calabrese G.C., Tricerri M.A. (2017). Learning from Synthetic Models of Extracellular Matrix; Differential Binding of Wild Type and Amyloidogenic Human Apolipoprotein A-I to Hydrogels Formed from Molecules Having Charges Similar to Those Found in Natural GAGs. Protein J..

[35] Townsend D., Hughes E., Hussain R., Siligardi G., Baldock S., Madine J., Middleton D.A. (2017). Heparin and Methionine Oxidation Promote the Formation of Apolipoprotein A-I Amyloid Comprising α-Helical and β-Sheet Structures. Biochemistry.

[36] Tanaka M., Kawakami T., Okino N., Sasaki K., Nakanishi K., Takase H., Yamada T., Mukai T. (2018). Acceleration of amyloid fibril formation by carboxyl-terminal truncation of human serum amyloid A. Arch. Biochem. Biophys..

[37] Semenyuk P., Muronetz V. (2019). Protein Interaction with Charged Macromolecules: From Model Polymers to Unfolded Proteins and Post-Translational Modifications. Int. J. Mol. Sci..

[38] Sorokina S.A., Stroylova Y.Y., Shifrina Z.B., Muronetz V.I. (2016). Disruption of Amyloid Prion Protein Aggregates by Cationic Pyridylphenylene Dendrimers. Macromol. Biosci..

[39] Sorokina S., Semenyuk P., Stroylova Y., Muronetz V., Shifrina Z. (2017). Complexes between cationic pyridylphenylene dendrimers and ovine prion protein: Do hydrophobic interactions matter?. RSC Adv..

[40] Klajnert B., Cortijo-Arellano M., Cladera J., Bryszewska M. (2006). Influence of dendrimer’s structure on its activity against amyloid fibril formation. Biochem. Biophys. Res. Commun..

[41] Radko S.P., Khmeleva S.A., Mantsyzov A.B., Kiseleva Y.Y., Mitkevich V.A., Kozin S.A., Makarov A.A. (2018). Heparin Modulates the Kinetics of Zinc-Induced Aggregation of Amyloid-β Peptides. J. Alzheimer’s Dis..

[42] Evstafyeva D.B., Izumrudov V.A., Muronetz V.I., Semenyuk P.I. (2018). Tightly bound polyelectrolytes enhance enzyme proteolysis and destroy amyloid aggregates. Soft Matter.

[43] Cohlberg J.A., Li J., Uversky V.N., Fink A.L. (2002). Heparin and Other Glycosaminoglycans Stimulate the Formation of Amyloid Fibrils from α-Synuclein in Vitro. Biochemistry.

[44] Mehra S., Ghosh D., Kumar R., Mondal M., Gadhe L.G., Das S., Anoop A., Jha N.N., Jacob R.S., Chatterjee D. (2018). Glycosaminoglycans have variable effects on α-synuclein aggregation and differentially affect the activities of the resulting amyloid fibrils. J. Biol. Chem..

[45] Barinova K.V., Kuravsky M.L., Arutyunyan A.M., Serebryakova M.V., Schmalhausen E.V., Muronetz V.I. (2017). Dimerization of Tyr136Cys alpha-synuclein prevents amyloid transformation of wild type alpha-synuclein. Int. J. Biol. Macromol..

[46] Izumrudov V.A., Zhiryakova M.V., Kudaibergenov S.E. (1999). Controllable stability of DNA-containing polyelectrolyte complexes in water–salt solutions. Biopolymers.

[47] Barinova K., Khomyakova E., Semenyuk P., Schmalhausen E., Muronetz V. (2018). Binding of alpha-synuclein to partially oxidized glyceraldehyde-3-phosphate dehydrogenase induces subsequent inactivation of the enzyme. Arch. Biochem. Biophys..

[48] Greenfield N., Fasman G.D. (1969). Computed circular dichroism spectra for the evaluation of protein conformation. Biochemistry.

[49] Bousset L., Pieri L., Ruiz-Arlandis G., Gath J., Jensen P.H., Habenstein B., Madiona K., Olieric V., Böckmann A., Meier B.H. (2013). Structural and functional characterization of two alpha-synuclein strains. Nat. Commun..

[50] Semenyuk P., Barinova K., Muronetz V. (2019). Glycation of α-synuclein amplifies the binding with glyceraldehyde-3-phosphate dehydrogenase. Int. J. Biol. Macromol..

